# Supplementary material for: The transition state and regulation of γ-TuRC-mediated microtubule nucleation revealed by single molecule microscopy
Source: eLife. 2020 Jun 15;9:e54253. doi: 10.7554/eLife.54253 (PMC7338055; doi:10.7554/eLife.54253)
Supplement: Transparent reporting form [file elife-54253-transrepform.pdf]

eLIFE
1st Floor
24 Hills Road
Cambridge CB2 1JP, UK
P 01223 855340
W elifesciences.org
T @elife

eLife's transparent reporting form

We encourage authors to provide detailed information *within their submission* to facilitate the interpretation and replication of experiments. Authors can upload supporting documentation to indicate the use of appropriate reporting guidelines for health-related research (see EQUATOR Network), life science research (see the BioSharing Information Resource), or the ARRIVE guidelines for reporting work involving animal research. Where applicable, authors should refer to any relevant reporting standards documents in this form.

If you have any questions, please consult our Journal Policies and/or contact us: editorial@elifesciences.org.

Sample-size estimation

- You should state whether an appropriate sample size was computed when the study was being designed
- You should state the statistical method of sample size computation and any required assumptions
- If no explicit power analysis was used, you should describe how you decided what sample (replicate) size (number) to use

Please outline where this information can be found within the submission (e.g., sections or figure legends), or explain why this information doesn't apply to your submission:

Number of experiments performed and measurements made (sample size) are detailed in the figure legend for the corresponding figure panel.

No sample size calculation was performed. Sample sizes were chosen with the following rationale for individual experiments.

For γ-TuRC nucleation assay in Fig. 1 and related supplements, the experiments with unlabeled γ-TuRC were repeated more than ten times with independent γ-TuRC preparations, while those fluorescent γ-TuRC repeated were repeated six times with three independent γ-TuRC preparations.

For reaction kinetics measurement from γ-TuRC in Figure 2 and related supplements, a set of reactions in vitro was performed using independent and fresh γ-TuRC purification each time. The time series was repeated identically three times as described in the figure legends and all data was analyzed. Representative nucleation-time curve is displayed and data from all replicates were pooled in the power-law analysis. The results from each replicate were found to be in agreement with the others.

For spontaneous nucleation assay in Figure 3A-B and related supplements, a set of reactions in vitro was performed using independent and fresh buffer each time. Experiments and analyses in were repeated thrice independently. The results from each replicate were found to be in agreement with the others. All data were pooled and reported. Direct, side-by-side comparison of γ-TuRC and spontaneous nucleation (Fig 3-supplement 1B-C), the experimental set and analyses in were repeated identically two times with independent γ-TuRC preparations. The results from the two replicates were found to be in agreement with each other.

For blunt seed-mediated microtubule assembly in Figure 3C-D and related supplemental figures, a set of reactions in vitro was performed using independent and fresh buffer each time. The time series was repeated three times as described in the figure legends and all data was analyzed. Representative nucleation-time curve is displayed and data from all replicates were pooled in the power-law analysis. The results from each replicate were found to be in agreement with the others.

For γ-/αβ-tubulin interaction assays, size exclusion runs in Figure 4A and Figure 4-supplement 1A were repeated three times, with the exception of 10μM αβ-tubulin run that was performed twice. For single molecule assays (Figure 4B-C), experiments and analyses in were repeated identically two times, pooled and reported. A third experimental set was performed where the observation began later at 180 seconds and was thus, not pooled. The results from each replicate were found to be in agreement with the others, including the results from the third replicate.

For Monte Carlo simulations in Figure 5 and related supplements and in Figure 7D-E, between 200 and 500 simulations were performed for a given tubulin concentration at every parameter set. Probability of nucleation versus time was plotted from these simulations, and power-law analysis was performed. All data is displayed. Transition state in Figure 5D was recorded for n=2119 simulations, all data was pooled and displayed in the histograms.

eLife Sciences Publications, Ltd is a limited liability non-profit non-stock corporation incorporated in the State of Delaware, USA, with company number 5030732, and is registered in the UK with company number FC030576 and branch number BR015634 at the address 1st Floor, 24 Hills Road, Cambridge CB2 1JP | August 2014

For assaying the effect of γ-TuNA, NME7 and TPX2 in Figure 6 and related supplements, experiments and analyses in were individually repeated twice with independent γ-TuRC preparations for γ-TuNA, twice on different days of experimentation with the same γ-TuRC preparations for NME7, and thrice with independent γ-TuRC preparations for TPX2. To pool and display all data, number of MTs nucleated in control reactions at a specific was set to 1 to account for variable γ-TuRC concentration across purifications. All data was pooled and reported. Individual datasets with ±γ-TuNA, ±NME7 and ±TPX2 is represented with solid or dashed curves.

For assaying the effect of XMAP215 on γ-TuRC mediated nucleation in Figure 7A-B, experiments and analyses in were repeated thrice with independent γ-TuRC preparations. To pool and display all data, number of MTs nucleated in control reactions at a specific was set to 1 to account for variable γ-TuRC concentration across purifications. All data was pooled and reported. Individual datasets with ±XMAP215, is represented with solid or dashed curves.

For reaction kinetics measurement from XMAP215 and γ-TuRC in Figure 7C-E and related supplements, a set of reactions in vitro was performed using independent and fresh γ-TuRC purification each time. The time series was repeated identically three times for all concentration points, and fewer concentration points were repeated another two times. All data was analyzed. Representative nucleation-time curve is displayed. All five datasets from all replicates were pooled and reported in the power-law analysis. The results from each replicate were found to be in agreement with the others.

For assaying the effect of MCAK and Stathmin in Figure 7F and related supplements, experiments and analyses in were individually repeated thrice individually for both MAPs with independent γ-TuRC preparations. To pool and display all data, number of MTs nucleated in control reactions at a specific was set to 1 to account for variable γ-TuRC concentration across purifications. All data was pooled and reported. Individual datasets with ±MCAK, ±Stathmin is represented with solid or dashed curves.

eLife Sciences Publications, Ltd is a limited liability non-profit non-stock corporation incorporated in the State of Delaware, USA, with company number 5030732, and is registered in the UK with company number FC030576 and branch number BR015634 at the address 1st Floor, 24 Hills Road, Cambridge CB2 1JP | August 2014

Replicates

- You should report how often each experiment was performed
- You should include a definition of biological versus technical replication
- The data obtained should be provided and sufficient information should be provided to indicate the number of independent biological and/or technical replicates
- If you encountered any outliers, you should describe how these were handled
- Criteria for exclusion/inclusion of data should be clearly stated
- High-throughput sequence data should be uploaded before submission, with a private link for reviewers provided (these are available from both GEO and ArrayExpress)

Please outline where this information can be found within the submission (e.g., sections or figure legends), or explain why this information doesn't apply to your submission:

The information on sample size and replicates are provided for each individual figure panel in the corresponding figure legend. A summary on the number of replicates for each experiment is also provided above. No data or outliers were excluded from the analyses.

Statistical reporting

- Statistical analysis methods should be described and justified
- Raw data should be presented in figures whenever informative to do so (typically when N per group is less than 10)
- For each experiment, you should identify the statistical tests used, exact values of N, definitions of center, methods of multiple test correction, and dispersion and precision measures (e.g., mean, median, SD, SEM, confidence intervals; and, for the major substantive results, a measure of effect size (e.g., Pearson's r, Cohen's d)
- Report exact p-values wherever possible alongside the summary statistics and 95% confidence intervals. These should be reported for all key questions and not only when the p-value is less than 0.05.

Please outline where this information can be found within the submission (e.g., sections or figure legends), or explain why this information doesn't apply to your submission:

Experiments
The number of measurements for all experimental data displayed in the paper were greater than 10. Nevertheless, we displayed individual data points or time-series in the individual plots in Fig. 2B-D, Fig. 3B, Fig. 3D-E, Fig. 5B-C, Fig. 6B,D, Fig. 7B, Fig. 7D-F as well as in related supplements. For growth speed curves (Fig. 2B and Fig. 3-supplement 1D), individual data points are plotted, and linear fit with shaded mean ± 2SD is displayed to represent the 95% confidence interval. For nucleation-time curves (Fig. 2C, Fig. 3D and supplement 1C, Fig. 6B,D, Fig. 7B,D,F and supplement 1B,E), individual time trace of number of MTs (m) vs time (t) is displayed. Shaded regions represent 95% confidence interval $(m \pm 2\sqrt{m})$ in the number of nucleated MTs (m) assuming a Poisson distribution as detailed in Methods. For power-law analysis curves (Fig. 2D, Fig. 3B,E, Fig. 7E), individual data points are displayed, and 95% confidence interval of the linear fit is reported on the plot. For Fig. 4C, number of bound molecules were counted as described in Methods. n=56 data points each were displayed as mean ± std in the bar graph.

Simulations
For Fig. 5-supplement 1A, 20 growth speed measurements were made at each tubulin concentration. Individual data points are plotted, and compared with experimental data. For Fig 5-supplement 1B(i),1C(i), Fig 5-supplement 2A(i),2B(i), cumulative probability distribution were obtained from 200-500 simulations. Individual time series is plotted. For power-law analysis curves (Fig. 5C, Fig 5-supplement 1B(ii),1C(ii), Fig 5-supplement 2A(ii),2B(ii)), individual data points are displayed, and 95% confidence interval of the linear fit is reported on the plot. For Fig. 5D, a histogram from n=2119 simulations is displayed. Mean ± SD value is reported for the histogram on the left.

The plotting scheme is detailed in the individual figure legends.
Statistical methods are reported and the values displayed (mean ± 2SD, mean ± SD, 95% confidence intervals) are described clearly in the figure legends for each panel displayed.

(For large datasets, or papers with a very large number of statistical tests, you may upload a single table file with tests, Ns, etc., with reference to sections in the manuscript.)

Group allocation

- Indicate how samples were allocated into experimental groups (in the case of clinical studies, please specify allocation to treatment method); if randomization was used, please also state if restricted randomization was applied
- Indicate if masking was used during group allocation, data collection and/or data analysis

Please outline where this information can be found within the submission (e.g., sections or figure legends), or explain why this information doesn't apply to your submission:

Samples were not required to be allocated into experimental groups. Various protein concentrations in vitro were changed and identical experiments were performed after changes in the protein concentrations. This information therefore does not apply to this submission.

Additional data files ("source data")

- We encourage you to upload relevant additional data files, such as numerical data that are represented as a graph in a figure, or as a summary table
- Where provided, these should be in the most useful format, and they can be uploaded as "Source data" files linked to a main figure or table
- Include model definition files including the full list of parameters used
- Include code used for data analysis (e.g., R, MatLab)
- Avoid stating that data files are "available upon request"

Please indicate the figures or tables for which source data files have been provided:

Time-series data generated in this study is directly plotted for all replicates. In the cases where time-series for several tubulin concentrations needed to be displayed on one plot, it was not possible to effectively represent the time-series from every replicate as that resulted in too many curves on one plot. In this case, we display a representative experiment and data from all replicates was pooled and reported for power-law analysis. Individual data points and time-series curves are directly plotted.

Source data

Source data for Figures 2B, 2C, 2D, 3-supplement 1C, 3-supplement 1D, 3B, 3D, 3E, 4C, 6B, 6D, 7B, 7D, 7E, 7F, 7-supplement 1B, 7-supplement 1E are provided in the excel sheet.

Code

For data analysis, no MATLAB software was generated and only inbuilt MATLAB functions were used. MATLAB software was developed to model γ-TuRC mediated nucleation with stochastic Monte Carlo simulations. Source code is provided in the supplementary materials and its documentation is provided in the Methods section.
